# Latest Advances in Chondrocyte-Based Cartilage Repair

**DOI:** 10.3390/biomedicines12061367

**Published:** 2024-06-19

**Authors:** Li Yue, Ryan Lim, Brett D. Owens

**Affiliations:** 1 Department of Orthopaedics, Rhode Island Hospital and Warren Alpert Medical School of Brown University, Providence, RI 02903, USA; brett_owens@brown.edu; 2 Department of Biology, Brown University, Providence, RI 02912, USA; ryan_lim@brown.edu; 3 University Orthopedics, East Providence, RI 02914, USA

**Keywords:** chondrocyte, cell therapy, cartilage repair, Cartibeads, Quantum hollow-fiber bioreactor, signaling pathway

## Abstract

Chondrocyte-based cell therapy has been used for more than 30 years and is still considered to be a promising method of cartilage repair despite some limitations. This review introduces the latest developments of four generations of autologous chondrocyte implantation and current autologous chondrocyte products. The regeneration of cartilage from adult chondrocytes is limited by culture-induced dedifferentiation and patient age. Cartibeads is an innovative three-step method to produce high-quality hyaline cartilage microtissues, and it is developed from adult dedifferentiated chondrocytes with a high number of cell passages. In addition, allogeneic chondrocyte therapies using the Quantum hollow-fiber bioreactor and several signaling pathways involved in chondrocyte-based cartilage repair are mentioned, such as WNT signaling, the BMP-2/WISP1 pathway, and the FGF19 pathway.

## 1. Introduction

Cartilage is a specialized form of flexible connective tissue and has limited capacity for regeneration and self-repair after being damaged due to its low cell density, and alymphatic and avascular nature [1]. Articular cartilage is made up of 70–80% water; 1–10% chondrocytes; 7–9% proteoglycan molecules, that give cartilage its shock-absorber quality; and 12–14% collagen, which are the strong fibers that hold it all together and resist tearing [2]. Cartilage injury and degradation affect over 250 million people worldwide, costing 1–2.5% of the gross domestic product in developed countries [3], and will affect over 350 million people worldwide by 2030 according to the World Health Organization report [4,5]. Science Daily demonstrated on November 30, 2022 that conditions that cause cartilage degeneration cost the US public health system more than USD 303 billion per year. Patients with cartilage injury and degradation experience increased pain and discomfort, leading to disability and a decrease in quality of life [6]. More than 250,000 cartilage repair surgeries are performed in the United States annually [7]. PR Newswire reported that the global cartilage repair/cartilage regeneration market in terms of revenue was estimated to be worth USD 1.3 billion in 2023 and is poised to reach USD 2.8 billion by 2028. Cartilage is composed of only one type of cell, known as a chondrocyte, which plays a crucial role in the process of cartilage formation, growth, repair, and remodeling. Chondrocytes are responsible for producing collagen and the extracellular matrix that maintains the cartilaginous tissues within joints and makes cartilage strong yet flexible [8]. There are new tissue engineering approaches, cell-based therapies, and advanced methods now being used for cartilage repair. This review introduces the latest advances in chondrocyte-based cartilage repair as follows.

## 2. Methodology

We searched the Google Scholar, PubMed, and Web of Science databases for articles published from their inception to 1 June 2024. We used the search keywords as follows: “cartilage repair”, “cartilage injury”, “advanced chondrocyte therapy”, “autologous chondrocyte therapy”, and “allogeneic chondrocyte therapy”. We included mainly articles published in the English language. Among these methods, we chose to introduce autologous chondrocyte implantation, therapy with a combination of pure chondrocytes and different chondrogenic cells (autologous or allogeneic), methods to produce high-quality hyaline cartilage microtissues, and increase cartilage mineralization and chondrogenesis.

## 3. Development of Autologous Chondrocyte Implantation

Articular cartilage is an avascular tissue and easily injured, and due to poor self-renewal, the transplantation of autologous cultured chondrocytes is the most commonly used cell-based therapy for human cartilage repair. Autologous chondrocyte implantation (ACI) is a two-stage advanced surgical procedure that uses the patient’s own chondrocytes to treat isolated areas of cartilage defects in the knee [9]. In ACI, a small piece of a patient’s cartilage is removed from the knee, and then the chondrocytes can divide and proliferate when they are separated from their matrix. Chondrocytes are grown, either in vivo or in vitro, until millions of cells are obtained. Cultured chondrocytes are then implanted into the damaged area of articular cartilage as a patch [10]. ACI can provide an alternative cell resource with a higher regenerative capacity for cartilage regeneration. ACI was first successful in an animal model in 1984 [11]. The first clinical use of autologous cultured chondrocytes for cartilage repair was in Gothenburg, Sweden, in 1987 by Lars Peterson and colleagues [11]. Then, ACI began to be regularly performed in humans in the mid-1990s [12], with the first pilot study published in 1994 [11]. Currently, ACI has become a worldwide well-established surgical technique and has been used to treat cartilage defects in thousands of patients. In 2021, four generations are developed during the evolution of the surgical techniques for ACI procedures over the past 30 years (Table 1). The evolution of ACI therapy is detailed below. 

(1) First-generation ACI: The biopsies of healthy cartilage are harvested from a minor load-bearing area of the knee, then the cartilage slices are sent to a lab for cell isolation and culture. The isolated chondrocytes are expanded in vitro for several weeks. The expanded and increased amount of chondrocyte suspension is injected into the defect, which is covered with a membrane of periosteum [11].

**Table 1:** Characteristics of four generations of ACI.

ACI Generation	Key Features	Advantages	Disadvantages
First	Chondrocyte suspension is injected under a membrane of periosteum. Product example: ChondroCelect^®^	For large lesions, it is an effective treatment and improves clinical outcomes compared to the microfracture repair technique.	They include leakage and inhomogeneous distribution of the injected chondrocytes, donor site morbidity, complexity of the surgical procedure, being highly invasive, and weak biomechanical properties. The occurrence of complications, such as periosteal graft hypertrophy, is frequent. Invasiveness is increased because of periosteal harvest and hypertrophy.
Second	Chondrocyte suspension is injected under a collagen membrane. Product example: BioCartTM II	Using a bioabsorbable collagen membrane instead of a membrane of periosteum addresses the shortcomings of first-generation ACI. It uses chondroprogenitor cells instead of pure articular chondrocytes to reduce donor site morbidity. These technique changes decrease graft hypertrophy and surgery-associated morbidity, and improve long-term clinical outcomes. It is more cost-effective than first-generation ACI.	They are long rehabilitation, potential surgical morbidity, invasiveness of the transplantation procedure, limitation of patient age, and fibrous tissue formation. There are high laboratory costs for cell expansion.
Third	Chondrocytes are grown on a surface carrier or in a matrix/scaffold. It is developed from a monolayer distribution of the cells to a 3-dimensional matrix/scaffold. Product examples: MACI^®^ and Chondron^®^	They are biocompatibility, homogeneous distribution of chondrocytes, less operative and hemostasis times, simple production process, and suitability for large cartilage defects. It facilitates minimally invasive transplantation and can be generated in various sizes and shapes. The use of matrix-induced chondrocyte implantation conquers the limitations of the first- and second-generation ACI.	It requires multiple operations with an open incision. The patients need a longer time to return to activity after the procedure. There is a risk of post-operation joint infection. The number of autologous chondrocytes may be limited for older patients or patients with serious diseases.
Fourth	Chondrocytes are implanted in different ways as a one-stage procedure mixing chondrocytes and bone marrow cells, without cell culture; the mixed cells are then seeded into a scaffold. Product example: Cartilife^®^	It consists of variants of particulated or minced allogeneic or autologous cartilage on scaffolds.	It is not successful every time. Some patients are poor responders to local biological repairs using this generation of ACI.

Clinical outcomes after first-generation ACI over a 20-year follow-up reported that 93% of twenty-three patients (24 knees) showed good to excellent clinical results, and the outcomes for 9 of 24 knees were considered failures. Overall, 79% of 23 patients maintained their native knee, and were satisfied when evaluated 20 years later [13]. The reason for post-operative failures was periosteal hypertrophy, in which overgrowth of the periosteum typically requires surgical intervention to shave the graft, or delamination of the periosteal tissue [13,14].

(2) Second-generation ACI: a collagen membrane is fixed to the surrounding cartilage to create a reservoir, and a suspension of culture-expanded autologous chondrocytes is injected under a collagen membrane [15,16]. Instead of a periosteum patch, use of the bioabsorbable collagen membrane avoids the frequent complication of graft hypertrophy, the requirement for surgical chondroplasty of the hypertrophic graft, and reduces the chances of graft delamination [17]. Clinical outcomes are similar to first-generation ACI, but second-generation ACI has fewer chances of revision surgery and could decrease the incidence of graft hypertrophy to 6% [18]. A statistically significant improvement was observed in all scores from the basal evaluation up to the 7-year follow-up for 62 patients (48 male and 14 female). A total of 11% of these 62 patients failed. A better outcome was obtained in young active men. The results suggest that second-generation ACI with the proper indications may offer good and stable clinical outcomes over time [19]. After a minimum of 10 years of follow-up, the results from 23 patients were compared to those of 23 matched patients who were administrated with first-generation ACI and demonstrated that second-generation ACI resulted in superior clinical long-term outcomes. Thus, second-generation ACI should be preferred over first-generation ACI [20].

(3) Third-generation ACI: Chondrocytes are grown on a surface carrier [21] or cells are seeded on or in a scaffold [22,23]. This generation of ACI is developed from a monolayer distribution of the cells to the 3-dimensional scaffolds or matrices. For young patients with large, locally restricted cartilage defects, third-generation matrix-supported ACI with various scaffolds showed good mid- to long-term outcomes. The Igor scaffold is a collagen matrix seeded with cultured autologous chondrocytes. Two-year follow-up of the clinical outcome and radiological scores indicated good to excellent results for most of the 21 patients (12 male and 9 female) who were treated with third-generation ACI using the Igor scaffold. A total of 19% of these 21 patients failed [24]. Furthermore, the data from 84 patients with full-thickness cartilage defects in the knee joints (41 athletic persons and 43 non-athletic persons) demonstrated that third-generation ACI is an appropriate treatment for athletic patients as compared to non-athletic patients [22]. Third-generation ACI using matrix-induced chondrocyte implantation conquers the limitations of the first and second generations. The advantages of third-generation ACI include reducing operative and hemostasis times more than those of conventional therapies, and facilitating minimally invasive transplantation [25]. 

(4) Fourth-generation ACI: In order to better replicate the innate cartilage prior to implantation, a fourth generation of ACI has been developed. This generation of ACI consists of variants of particulated or minced allogeneic or autologous cartilage on scaffolds, such as new forms of microfracture, gel-type ACI, cartilage implantation, single-stage procedures, other cell type usage, metal or plastic patches for knees, etc. [12]. In fourth-generation ACI, chondrocytes are implanted in different ways as a one-stage procedure, mixing chondrocytes and bone marrow cells, without cell culture, the mixed cells are then seeded into a scaffold, which is then implanted into the cartilage defects. The combination of chondrocytes and bone marrow mesenchymal stem cells (autologous or allogeneic) are seeded in a matrix [26,27]. In another development, the new cartilage was implanted after it grew from the autologous chondrocytes in vitro, in place of implanting cultured chondrocytes into the cartilage defect [28]. Gel-type ACI, with the use of cells held in place with fibrin, is a new variant without the use of a membrane or periosteum, and gel-type ACI has been shown to be a secure and effective method for both relieving pain and improving knee function [29]. Cartilage progenitor cells derived from ear elastic cartilage could be useful for reconstructing joint hyaline cartilage [30]. Another development is that instead of a chondrocyte cell culture, hyaline cartilage is minced after harvest and attached to a biodegradable fibrin glue scaffold in a single-stage procedure [31].

In the last twenty years, eight autologous chondrocyte products have been approved worldwide: three European products (ChondroCelect^®^ in 2009 [32,33], MACI^®^ in 2013 [34], and Spherox^®^ in 2017 [35,36]), two Korean products (Chondron^®^ in 2001 [29,37] and Cartilife^®^ in 2019 [37,38]), one US product (MACI^®^ in 2016 [39]), one Australian product (Chondrocytes-T-Ortho-ACI^®^ in 2017 [38]), and one Japanese product (JACC^®^ in 2012 [38,40]). ChondroCelect^®^ and MACI^®^ were approved in the European Union and removed from the market. Cartilife^®^, Chondrocytes-T-Ortho-ACI^®^, Spherox^®,^ and JACC^®^ are currently undergoing additional safety investigations as part of post-marketing surveillance [38]. Within these products, Cartilife^®^ is a fourth-generation ACI in which donor tissue is collected from the rib cartilage of a patient, and then cultured to generate pellets that contain cartilage cells and extracellular substrates, finally, pellet-type chondrocyte tissue is implanted into the defective area without a scaffold [38]. Chondrocytes-T-Ortho-ACI^®^ utilizes chondrocytes seeded onto a collagen scaffold after healthy chondrocytes are cultured [38]. Spherox^®^ is a matrix-associated, three-dimensional ACI used to treat knee cartilage defects. Spherox^®^ is a product in which human chondrocytes are cultured in vitro in a monolayer, and then, transplanted into the extracellular matrix, leading to three-dimensional spheroids [36]. JACC^®^ is an autologous chondrocyte in which the patient’s own cartilage tissue is harvested and mixed with atelocollagen gel to form a cartilage-like tissue when cultured in a three-dimensional environment, then the cartilage-like tissue is transplanted at the site of cartilage-defective area. The approval for JACC^®^ was partially modified in 2019 to further reduce the invasiveness of JACC^®^ treatment for patients, using an artificial collagen film instead of periosteum to minimize invasive transplant procedures [38]. The therapy medicinal products for autologous chondrocytes are listed in Table 2.

Until now, chondrocyte-based therapy is still considered to be a promising way of repairing cartilage damage because chondrocytes are the most natural cells and are valuable to use for cartilage repair due to their reconstructive nature [41]. Instead of pure chondrocytes, different chondrogenic cells could be used for cartilage repair, such as bone marrow mesenchymal stem cells [42], muscle-derived stem cells [43], adipose-derived stem cells [44], peripheral blood stem cells [45], synovial membrane-derived stem cells [46,47], and menstrual blood-derived mesenchymal stem cells [48]. These adult stem cells can be induced to differentiate into chondrocytes and could be utilized for cartilage repair. 

The advantage of an ACI procedure is that the chondrocytes are harvested from the patient’s own cartilage; therefore, the risk of a graft being rejected by the patient’s own body is decreased. The disadvantage is ACI requires an open incision, the number of expanded chondrocytes are limited in a short-term culture, and expansion differentiation of chondrocytes from a population of older patients is difficult. Allogeneic chondrocyte therapies using a Quantum hollow-fiber bioreactor may provide another treatment option to obtain a large number of chondrocytes from optimal healthy donors in a short-term culture compared to autologous cells. In order to engineer high-quality hyaline cartilage microtissues, called Cartibeads, an innovative method has been developed from adult dedifferentiated chondrocytes with a high number of cell passages. 

## 4. Cartibeads

Chondrocyte-based cell therapies for repair of cartilage defects have been used for over 25 years despite current limitations. Chondrocyte dedifferentiation is a major obstacle for effective cell-based cartilage repair and regeneration. In addition, chondrocyte dedifferentiation causes inferior repaired tissues with dysfunctional fibrocartilage in vivo [25] and has severely compromised the clinical outcomes of cartilage repair [41]. The mechanisms of chondrocyte dedifferentiation may be due to epigenetic factors [49,50], oxidative stress [51], senescence [51] and the mechanical microenvironment [52,53], DNA methylation [49], regulation of insulin-like growth factor binding protein-3, intercellular adhesion molecule-1, vascular cell adhesion molecule-1, vascular endothelial growth factor, transforming growth factor β2 [54], extracellular-signal-regulated kinase (ERK), and bone morphogenetic protein (BMP)-2 signaling [55]. Hyaline cartilage is characterized by a matrix containing glycosaminoglycan and type II collagen. The role of chondrocyte–matrix interactions is maintaining and repairing articular cartilage [56]. In order to obtain sufficient numbers of human chondrocytes for implantation, chondrocyte expansion is required after biopsy. However, adult chondrocytes gradually lose function and phenotype, dedifferentiating into a fibroblast-like morphology during the expansion process in monolayer culture, which leads to the inability to produce glycosaminoglycan and type II collagen. Chondrocyte dedifferentiation usually occurs at the second or third cell passage [56,57]. Differentiated chondrocytes are fibroblast-like cells that produce type I collagen, a primary component of fibrocartilage. Hyaline cartilage is made up primarily of type II collagen only, whereas fibrocartilage is composed of type I and II collagen. Fibrocartilage is a mixture of hyaline cartilage and dense connective tissue [58]. Fibrocartilage is not an effective tissue for long-lasting cartilage repair due to its being biomechanically different from hyaline cartilage [59]. There is another limitation for autologous chondrocyte-based cartilage repair. This limitation is that expansion differentiation of chondrocytes from populations of older patients is difficult [60].

An innovative three-step method to engineer high-quality hyaline cartilage microtissues, called Cartibeads, was developed from adult dedifferentiated chondrocytes with a high number of cell passage [61,62]. It was reported that Cartibeads engineered from chondrocytes are capable of treating cartilage lesions through complete fusion of Cartibeads among themselves and their integration with the surrounding native cartilage and subchondral bone [61]. Human cartilage samples were harvested (from ages 18 to 80 years) and cut into 1 mm small pieces. Then, the cartilage pieces were digested with collagenase type II. Once extracted, cells were washed and cultured in a 2-dimensional monolayer culture (passage 0). At confluence, the cells were seeded in one flask (passage 1) and later split into two flasks (passage 2) until reaching confluence. At this stage, cultured chondrocytes could be frozen for back-up. After cell proliferation (step 1), chondrocytes were re-differentiated for 7 days (step 2). Cell growth was reduced in step 2 (the re-differentiation phase). In step 3, chondrocytes were harvested and resuspended in chondrogenic medium to obtain 0.2 × 10^6^ cells/well (1 Cartibeads per well) in 96-well polypropylene plates (~20 × 10^6^ cells/plate). After 15 days in 3-dimensional culture, the 96-well plates were then centrifuged for 5 min to allow cell aggregation into Cartibeads. Then, Cartibeads were taken out from the 96-well plates and pooled together. Chondrocytes used for Cartibead formation in the two- and three-step methods came from passage 3 to 9 chondrocytes [61,62]. Preclinical safety studies showed that Cartibeads were not tumorigenic when transplanted into mice [62]. The expressions of the WNT5A, WNT5B, and WNT7B genes were higher in dedifferentiated chondrocytes, and a decrease in the WNT signaling pathway promoted chondrogenic re-differentiation, producing an extracellular matrix with characteristics similar to hyaline cartilage [62]. Cartibeads are an innovative three-step method that is capable of producing hyaline-like cartilage microtissues from a small cartilage biopsy, regardless of the donor age and cartilage harvest quality, and Cartibeads could be a promising candidate for cartilage repair.

## 5. Allogeneic Chondrocyte Therapies Using Quantum Hollow-Fiber Bioreactor

ACI uses autologous cultured chondrocytes to treat articular cartilage defects of the knee by implanting the patient’s own cartilage cells. Autologous chondrocyte therapies are limited by the number of expanded chondrocytes at passages 2–3. Also, the ACI technique is a two-step surgical procedure: arthroscopy followed by an arthrotomy. Allogeneic chondrocytes can be cultured from healthy donors. These healthy donors can be selected by the high quality of their donor cartilage tissue and the chondrocytes can be extracted from this cartilage. Allogeneic chondrocyte therapy has the potential to produce large amounts of high-quality chondrocytes. Furthermore, allogeneic chondrocyte therapy only needs a single-stage surgical procedure as opposed to the two-stage ACI procedure. Therefore, allogeneic chondrocyte therapies may offer another treatment option to allow more individuals to be treated with chondrocyte therapies for cartilage defects. Allogeneic chondrocyte therapies can also reduce the cost burden of the current autologous ACI surgical procedures [63]. 

It was reported that large numbers of adult chondrocytes can be manufactured by the Quantum^®^ cell expansion system using a compliant hollow-fiber bioreactor. The Quantum bioreactor may provide a proper cartilage repair technique for developing allogeneic chondrocyte therapies [63]. Chondrocytes were taken from cartilage specimens of five patients who were undergoing total knee replacement for the treatment of end-stage osteoarthritis. Then, chondrocytes were seeded in the human platelet lysate to ensure that sufficient chondrocytes were obtained at passages 0 and 1. The Quantum cell expansion system was pre-coated with pooled human cryoprecipitate from five donors diluted 1:1 with PBS. The inner surface of the fibers was coated overnight with human cryoprecipitate solution before cell loading. This coating allows for the attachment of the cells to the polysulfone hollow fibers. After 5–10 million chondrocytes were placed into the Quantum bioreactor and left to adhere with uniform suspension, the Quantum cell expansion system was conditioned with human platelet lysate media and maintained perfusion of the human platelet lysate medium over the cells while an equal volume of conditioned medium was being removed. As the chondrocyte amount in the Quantum system increased, the perfusion rate of fresh medium was augmented 16-fold from a baseline rate of 0.1 mL/min to an inlet rate of 1 .6 mL/min. Lactate levels within the conditioned medium were measured daily using a Lactate Plus meter and a clinical blood glucose meter. Once the conditioned medium lactate and/or glucose concentration was evaluated and its growth rate was deemed to have plateaued, chondrocytes were collected [63,64]. It was found that significantly more chondrocytes were expanded in the Quantum cell expansion system as compared to traditional tissue culture plastic. There was no difference in growth kinetics, chondrogenic potential, cell morphology, or expression of chondropotency indicators (SOX9, CD39, CD44, CD151 and CD166) and mesenchymal stromal cell profile indicators (CD14, CD19, CD34, CD45, CD73, CD90 and CD105) between Quantum-expanded and traditional tissue culture plastic-expanded chondrocytes. Therefore, the Quantum bioreactor is a hollow-fiber system that could be an attractive technique to produce a larger number of chondrocytes for clinical use [63].

## 6. Signaling Pathways Involved in Chondrocyte-Based Cartilage Repair

### 6.1. BMP-2/WISP1 Signaling Pathway

A critical cranial defect in a rat model was repaired using WNT1-inducible-signaling pathway protein (WISP1)-pretreated chondrocyte scaffolds [65]. At first, the cartilage mineralization was induced in vitro using micromass primary chondrocyte cultures incubated with BMP2 and/or WISP1. Human chondrocytes were isolated from the articular cartilages of osteoarthritis patients undergoing total knee arthroplasty (*n* = 33, 14 males, 19 females, mean age was 73 years). Chondrocytes were cultured and passaged to reach passage 1 or 5. Passage 5 or passage 1 chondrocytes were then cultured in micromass cultures, while cells at passage 1 were additionally seeded into Zimmer^®^ Collagen Tape. Next, chondrocytes were seeded on collagen scaffolds in vitro. Finally, chondrocyte-seeded collagen scaffolds were used in a rat critical-sized calvarial defect model. The results indicated that the use of autologous chondrocytes repaired critical maxillofacial defects. Chondrocytes transplanted into critical cranial defects could accelerate the formation of native-like osseous tissue, especially after WISP1 treatment in cultured cells [65]. 

Mineralization of cartilage is critical for the formation and stability of bones [66] as well as skeletal tissues [67]. Cartilage provides a model for bone growth and mineralization. Sufficient mineralization is important for the construction and strengthening of bones and skeletal tissues [67]. With poor mineralization, there can be visible decreases in bone density that can cause osteoporosis, resulting in a higher likelihood of fractures [68,69]. Therefore, finding ways to increase cartilage mineralization would promote bone formation and density, leading to greater bone integrity. 

Dvir-Ginzberg et al. explored the effects of utilizing both BMP-2 and WISP1 in cartilage to promote mineralization [65]. BMP-2 and WISP1 are both factors known to increase osteogenesis. BMP-2 is known to help with osteogenesis by reducing cartilage callus formation, facilitating osteogenesis initiation, and progressing cartilage into the mineralization phase with improved physical properties [70,71,72,73,74]. BMP-2 is essential for chondrocyte maturation and endochondral ossification at the beginning of skeletal development [70]. WISP1 also contributes to osteogenesis through the promotion of mesenchymal cell proliferation, repression of chondrocytic differentiation, and facilitation of osteoblastic differentiation [75]. Young et al. discovered that WISP1 displayed a positive influence on bone cell differentiation and function. From the data, they hypothesized that increasing BMP-2 levels with increased WISP1 expression would result in increased osteogenesis [76]. Dvir-Ginzberg et al. expanded on this study by culturing cells from osteoarthritis donors with BMP-2 and WISP1 to determine cartilage mineralization. Compared with control groups, the data supported that high doses of BMP-2 or WISP1 can have positive influences on cartilage mineralization but certain concentrations of both yielded results similar to the controls. The authors noted that WISP1 treatment induced β-catenin in cell nuclei versus untreated controls. Similar to WISP1′s effects, BMP-2 treatment exhibited similar β-catenin in cell nuclei. This effect was evident in the combined treatments with low concentrations of WISP1 and BMP-2, which improved some aspects of cartilage mineralization and in vivo bone formation [65]. 

Some issues to note with this experiment lie with the downstream effects of WISP1 and the impacts of advanced passaging. WISP1 induces β-catenin, which is highly mutable and can result in various types of cancer [77]. Due to its carcinogenic nature, cells cultured with WISP1 were cultured carefully, and for the in vivo study, cells were pre-cultured with WISP1, and then, injected into rats that were analyzed [65]. In addition to troubles with WISP1, advanced passaging blocked the capacity of chondrocytes to mineralize when cultured in BMP-2 or WISP1 since advanced passaging impaired chondrocytic phenotypes and most likely harbored fibrotic cells [65,78]. The goal of Dvir-Ginzberg et al. was to use chondrocytes as an autologous source to support cranial fracture repair [65]. While co-treatment of adult chondrocytes with BMP-2/WISP1 did not enhance ossification, they were each able to individually increase mineralization. The increased mineralization is important for successful ossification and can potentially help with osteoporosis [66]. Previous studies have explored the effects of BMP-2 on osteoporosis. Data prove that BMP-2 prevents osteoporosis; however, it can lead to osteoclasts [79]. Research has proven that BMP-2/WISP1 co-treatment does not help ossification and further exploration must be determined to see how BMP-2/WISP1 co-treatment can affect osteoporosis. 

### 6.2. FGF19 Signaling Pathway

Fibroblast growth factor 19 (FGF19) is important for cartilage development and chondrogenesis [80]. Xie et al. aimed to explore FGF19-mediated cellular behavior in chondrocytes through mitochondrial biogenesis. It was established that mitochondria are essential for the survival and repair of cartilage due to their ability to generate adenosine triphosphate (ATP). Without properly functioning mitochondria, ATP generation is impaired, which directly interferes with the repair process for cartilage [80,81,82]. With increased mitochondria fusion, however, there can be elongation of the mitochondrial network under physiological conditions which can lead mitochondria to survive longer and support muscle homeostasis in aging [83], as well as preventing aging-related cartilage and joint diseases.

FGF19 plays an essential role in cartilage development and the physiology of cartilage [80,84]. Unclear on the process by which FGF19 influences cellular behavior in chondrocytes, Xie et al. explored how FGF19 influences mitochondrial biogenesis via AMP-activated protein kinase (AMPK)α-p38/mitogen-activated protein kinase (MAPK) signaling. The authors discovered the vital accessory protein for binding FGF19 to its receptor, β Klotho, helped FGF19 enhance mitochondrial biogenesis in chondrocytes. This increased biogenesis, along with the fusion of mitochondria, resulted in the elongation of mitochondria and an increase in mitochondrial fusion proteins [80,83]. In addition, FGF19 boosts the production of citrate synthase, which is crucial for aerobic respiration, which not only enhances mitochondrial biogenesis but also increases the number of functional mitochondria in chondrocytes [80]. The increased number of functional mitochondria boosts ATP production through oxidative phosphorylation [85] and helps improve programmed cell death, calcium homeostasis, tissue damage, innate immune response, intermediate metabolites oxidation, and cell homeostasis regulation [80,86,87,88].

The increase in mitochondrial biogenesis was mediated through the p38/MAPK signaling pathway. FGF19-mediated mitochondrial biogenesis mainly relies on the activation of phospho-p38 signaling, which increases MAPK signaling to promote mitochondrial biogenesis and fusion [80]. In general, MAPK have positive influences on cartilage development. Xu et al. discovered that MAPK is upregulated during craniofacial cartilage development in zebrafish [89,90]. The specific p38 MAPK plays multiple roles in chondrocyte differentiation [91,92,93]. It is considered an active regulator in chondrocyte differentiation, as well as chondrogenesis [90], due to its positive regulation of differentiation of prechondrogenic limb into hyaline chondrocytes which produce hyaline cartilage, the most widespread cartilage that is usually present within joints [94]. All of the positive influences FGF19 has on cartilage by increasing mitochondrial biogenesis and promoting chondrogenesis provide greater insight into the impacts FGF19 has on chondrocytes and cartilage. This expansion of our understanding of FGF19 promotes FGF19 as a potential therapeutic target for cartilage diseases [80].

## 7. Conclusions

**Figure 1:**
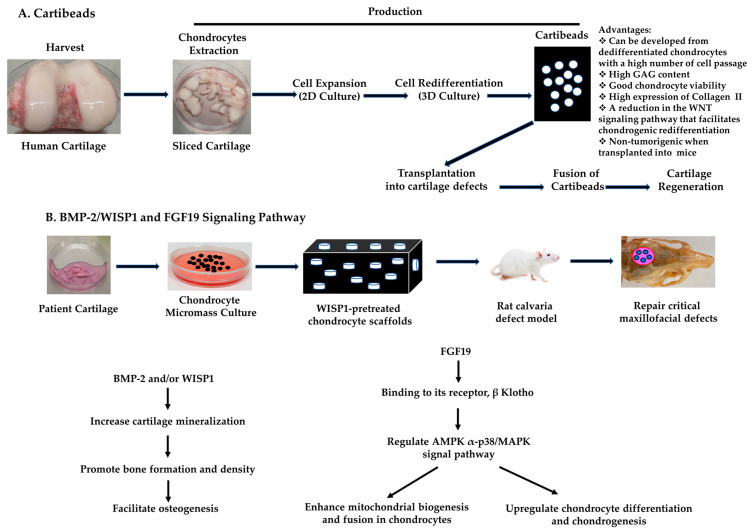
Schemes of the 3-step method for the generation of Cartibeads (**A**) and BMP-2/WISP1 and FGF19 signaling pathways (**B**).

Four generations of ACI have been developed over the past 30 years; however, the use of adult chondrocytes to regenerate cartilage is limited by culture-induced dedifferentiation, patient age, and the number of expanded chondrocytes within a short time. Allogeneic chondrocyte therapies can be cultured from optimal healthy donors and have the potential to produce large numbers of high-quality chondrocytes within a limited time. Allogeneic chondrocyte therapies could repair cartilage defects of the knees with larger defect sizes than ACI. Moreover, Cartibeads engineered from adult dedifferentiated chondrocytes with a high number of cell passages are capable of treating cartilage lesions. The Quantum hollow-fiber bioreactor could manufacture large numbers of adult chondrocytes. In the future, more innovative methods will be created to produce large numbers of adult chondrocytes for autologous or allogeneic chondrocyte therapies, and the underlying signal pathways involved in chondrocyte-based cartilage repair will be investigated. Schemes of the three-step method for the generation of Cartibeads and the BMP-2/WISP1 and FGF19 signaling pathways are shown in Figure 1.

## 8. Future Perspective

It was reported in 2022 that the autologous chondrocyte patch fabrication technique was efficient and cost-effective for four patients with knee joint cartilage defects. Two-year follow-up for clinical and radiological outcomes after patients undergoing fabrication via this sandwich technique showed clinical improvement and good quality of repaired cartilage tissue. However, there is a risk of a poor post-operative outcome for patients with osteoarthritis [95]. These results suggested that cost-effective strategies are critical for the potential efficient techniques and innovative approaches used for cartilage repair in patients. Large studies with long-term follow-up are needed to ensure the safety and efficacy of new methodologies.

In 2024, a study was the first to report the clinical outcomes of collagen-covered ACI using JACC^®^. A total of 69 patients with knee joint chondral defects who underwent ACI using JACC^®^ were investigated in this study. There were 34 patients using periosteum-covered ACI and 35 patients using collagen-covered ACI. No difference in post-operative subjective scores was observed between the periosteum-covered ACI and the collagen-covered ACI group [96]. The collagen-covered ACI group showed a lower adverse event rate and higher International Cartilage Repair Society Cartilage Repair Assessment scores. Using JACC^®^ in ACI, a collagen membrane was more effective, and decreased operation time and invasiveness [96].

Innovation strategies of fifth-generation ACI are being investigated, including cost reduction, short post-surgery rehabilitation, easy implantation, increases in cell proliferation and maturation, a decrease in surgical morbidity, enhancement in the formation and distribution of hyaline cartilage, maintenance of chondrocyte phenotype, and integration with the surrounding articular tissue.

## Figures and Tables

**Table 2:** Therapy medicinal products for autologous chondrocytes.

Product	ACI Generation	Implanted/Injected Body Zone	Advantages	Disadvantages	Price/Cost
ChondroCelect^®^ (Approved in European Union; withdrawn from the market in 2016 because of a reimbursement problem)	First	Implanted in femoral condyle of the knee.	ChondroCelect is more effective than microfracture at healing the defects in the cartilage, showing better structural repair.	Must be prepared specially for each individual patient and can only be used to treat the particular patient it was prepared for. May cause arthralgia (joint pain), cartilage hypertrophy, joint crepitation (unusual crackling sounds), arthrofibrosis, and swelling of the joint. Also, cannot be used by patients allergic to the ingredients or bovine serum, with advanced osteoarthritis of the knee, or with a femoral growth plate that is not fully closed.	GBP 17,740 for total procedure in United Kingdom
MACI^®^ (Approved in European Union; manufacturing plant in Europe closed due to low sales, so MACI is unavailable for new patients until a new manufacturing site has been registered)	Third	Implanted in damaged area of knee cartilage, held in place using fibrin sealant.	MACI is more effective than microfracture surgery at relieving pain and improving knee function.	Excess cartilage growth and detachment of the implant may occur in between 1 and 10 patients in 1000 treated with MACI. Other side effects revolve around surgical procedure risks. MACI cannot be used in patients with severe osteoarthritis, inflammatory joint disease, not fully closed growth plates in the thigh bone, or uncorrected inborn blood clotting disorders.	GBP 14,804 for total procedure in Germany Statutory Health Insurance System
Spherox^®^ (Approved in European Union; currently being subjected to additional safety investigations as part of post-marketing surveillance)	Third/fourth	During arthroscopy, injected into patient’s knee cartilage/extracellular matrix.	As effective as microfracture for small defects and stably improved large cartilage defects as well. Chondrocyte spheroids that attach to the knee cartilage allow for less invasive surgery.	The most common side effects of Spherox (which may affect up to 1 in 10 people) are bone marrow edema, arthralgia, joint effusion, swelling of the joint and pain. Also, cannot be used in patients with primary generalized/advanced osteoarthritis, growing knee joints, or with hepatitis B, C, and/or HIV.	GBP 10,000 per culture per patient in United Kingdom. Costs may vary in different settings because of negotiated procurement discounts
Chondron^®^ (Approved in South Korea)	Third	Injected into multiple holes drilled into the defect area.	The necessity of a second incision to harvest tibial periosteum can be avoided and the surgery time can be shortened. One vial of Chondron™ could cover a total condyle defect, allowing for a watertight cover and preventing the risk of breakdown of the treated lesion and its subsequent progression to arthritis.	Described as a safe and effective method for restoring patient knee function, though the benefits of fibrin may need to be further studied to justify the additional cost of fibrin.	Typically covered by insurance in South Korea
Cartilife^®^ (Approved in South Korea; currently being subjected to additional safety investigations as part of post-marketing surveillance)	Third/fourth	Injected into cartilage defect.	Potentially further shortens the rehabilitation period because side effects such as foreign body reactions are fewer, and there is no need to wait for the implanted cells to harden.	Currently still undergoing phase 3 clinical trials, as well as phase 2 clinical trials underway in the US for FDA approval. No adverse reactions have been reported as of yet.	Typically covered by insurance in South Korea
MACI^®^ (Approved in the United States of America)	Third	Implanted in damaged area of knee cartilage, held in place using fibrin sealant.	MACI is more effective than microfracture surgery at relieving pain and improving knee function.	Excess cartilage growth and detachment of the implant may occur in between 1 and 10 patients in 1000 treated with MACI. Other side effects revolve around surgical procedure risks. MACI cannot be used in patients with severe osteoarthritis, inflammatory joint disease, not fully closed growth plates in the thigh bone, or uncorrected inborn blood clotting disorders.	About USD 40,000, but insurance providers often cover most of those costs in the United States of America
Chondrocytes-T-Ortho-ACI^®^ (Approved in Australia; currently being subjected to additional safety investigations as part of post-marketing surveillance)	Third	Implanted in damaged area of knee cartilage using collagen scaffold.	Though with a limited number of cases, demonstrated good to excellent MRI and arthroscopic repair outcomes. Studies suggest that the procedure is safe, clinically effective, and represents an innovative and cost-effective ACI procedure.	May result in adverse outcomes such as graft overgrowth (hypertrophy) or loss of the graft (delamination), which may cause pain and restriction of function/movement. Also, is not recommended for use in patients outside of 18–65, severe osteoarthritis, inflammatory joint disease, allergies to gentamicin (antibiotic) or bovine serum, blood clotting disorders, or a compromised immune system.	Unknown, largely varies by insurance coverage in Australia
JACC^®^ (Approved in Japan; currently being subjected to additional safety investigations as part of post-marketing surveillance)	Third	Transplanted to a full-thickness cartilage defect in the knee, patched with collagen.	Unlikely to have a rejection reaction and can treat large knee defects. Prevents leakage of chondrocytes from the transplanted site, uneven distribution of chondrocytes, and decreased matrix productivity as compared to the monolayer culture. Reduces operation time and invasiveness due to collagen cover compared to autologous periosteum.	Evidence shows that most type II collagen was found to be present 30–60 months after treatment, suggesting that cartilage repair tissue produced following ACI treatment takes some years to mature. Also, potential problems such as the loss of critical chondrocytes caused by the cutting and repeated manipulation of the seeded membrane. There is also the possibility of detachment of the collagen membrane from the cartilage defect.	Covered by insurance in Japan
Novocart 3D^®^ (Currently in phase III clinical testing in the United States of America)	Third	Implanted in damaged area of knee cartilage.	Novocart 3D is expected to be more effective than microfracture at healing the defects in the cartilage, showing better structural repair. The scaffold design provides a more homogeneous distribution of cells and a layer of robust collagen membrane cover, allowing the chondrocytes to maintain shape and be protected after implantation.	Patients who were treated with Novocart 3D implants after an acute event (acute trauma or OCD) are at risk of developing a graft hypertrophy in the post-operative course of two years.	Not known at this time (not commercially available in the United States of America)
NeoCart^®^ (Currently in phase III clinical testing in the United States of America)	Third	Implanted in damaged area of knee cartilage.	NeoCart has been shown to significantly reduce pain within 6 months after treatment and shows trends toward improved function and motion, providing significantly greater improvements than microfracture. MRI indicated implant stability and peripheral integration, defect fill without overgrowth, progressive maturation, and more organized cartilage formation.	Though currently premature given the small number of patients in the completed trials, the treatment appears to be a safe and promising alternative to current restorative techniques for partial to full-thickness cartilage defects.	Not known at this time (not commercially available in the United States of America)
Agili-C™ (Approved in United States of America)	Third/fourth	Implanted in damaged area of knee cartilage.	Shown as more effective than microfracture for both small defects and large cartilage defects. Overall adverse event rates were lower than microfracture, supporting a very favorable safety profile.	The most common side effect with Agili-C is increased transient knee pain, though at a significantly lower rate than in microfracture. Agili-C cannot be used in patients with severe osteoarthritis, inflammatory joint disease, allergies to calcium/calcium-carbonate/coral, lacking healthy bone wall or inappropriate bone thickness, or bone disorders that may affect bone healing.	Though commercially available, price currently unknown (likely covered by insurance, though coverage varies in the United States of America)
BioCartTM II (Completed phase II clinical testing in the United States of America and Israel)	Second	Implanted in damaged area of knee cartilage using a mini-arthrotomy.	BioCart™II eliminates the need for a periosteal flap and enables implantation by a minimally invasive procedure, thus significantly simplifying surgery and reducing rehabilitation time. The porous open channel structure of the scaffold allows for an immediate three-dimensional distribution of the cells within the scaffold to promote full-thickness repair in a quick time frame, accelerating rehab and weight bearing.	Though currently premature given the small number of patients in the completed trials, the treatment appears to be a safe and promising alternative to current restorative techniques for partial to full-thickness cartilage defects.	Not known at this time (not commercially available in the United States of America or in Israel)
